# B-vitamin-associated miRNAs are enriched in pathways related to cell proliferation - insights from the general population

**DOI:** 10.1186/s12986-026-01177-2

**Published:** 2026-07-22

**Authors:** Sandra Van der Auwera, Sabine Ameling, Nele Friedrich, Matthias Nauck, Uwe Völker, Henry Völzke, Hans J. Grabe

**Affiliations:** 1 Department of Psychiatry and Psychotherapy, University Medicine Greifswald, Greifswald, Germany; 2 German Centre for Neurodegenerative Diseases (DZNE), Site Rostock/Greifswald, Greifswald, Germany; 3 Department of Functional Genomics, Interfaculty Institute for Genetics and Functional Genomics, University Medicine Greifswald, Greifswald, Germany; 4 DZHK (German Center for Cardiovascular Research), partner site North, Greifswald, Germany; 5 Institute of Clinical Chemistry and Laboratory Medicine, University Medicine Greifswald, Greifswald, Germany; 6 Institute for Community Medicine, University Medicine Greifswald, Greifswald, Germany

**Keywords:** Vitamin B12, Folic acid, miRNAs, Cell-cycle regulation, SHIP study, Red blood cells, Erythropoiesis

## Abstract

**Background:**

Vitamins are essential micronutrients that play crucial roles in human health, including immune function, cell development, energy metabolism, and the function of the nervous system. Within the body, vitamins can activate and modulate various biological pathways by acting as cofactors for enzymes. The objective of this study was to identify microRNAs (miRNAs) associated with the serum concentration of specific vitamins in the general population, and subsequently identifying vitamin associated biological systems and diseases potentially influenced by post-transcriptional regulation of these miRNAs.

**Methods:**

Data from the population-based *Study of Health in Pomerania* (SHIP-TREND-0, *N* = 792) were utilized to examine the associations between vitamin B12 and folic acid (vitamin B9) and 181 miRNAs. Multivariate linear models were employed, adjusting for potential confounders such as age, sex, platelet count, BMI, hemolysis, batch effects, and seasonality. Publicly available databases were used to identify biological pathways influenced by those significant miRNAs.

**Results:**

After correction for multiple comparisons, eight miRNAs were found to be positively associated with folic acid, and six miRNAs were positively associated with vitamin B12. The miRNAs significantly associated with folic acid were predominantly found in red blood cells, while those associated with vitamin B12 were associated with red blood cells and T-cells according to the miR-Blood database. Analyses of over-represented miRNAs revealed enrichment in processes related to cell-cycle regulation, DNA synthesis, metabolic processes, and erythropoiesis. In accordance to the known positive role of folic acid and vitamin B12 in pregnancy, nearly all significant miRNAs have been identified to be associated with pregnancy outcomes based on previous research.

**Conclusions:**

The analyses revealed that several miRNAs are related to vitamin levels and that these miRNAs are associated with processes such as cell proliferation and human fertility. These miRNAs could be interesting targets for further clinical applications.

**Supplementary Information:**

The online version contains supplementary material available at 10.1186/s12986-026-01177-2.

## Background

It is long known that nutrients and other bioactive food components are essential for immune function and disease prevention. Vitamins are organic micronutrients required in small amounts to support various biochemical processes essential to human health [1]. They function as cofactors or coenzymes in numerous metabolic pathways, influencing cellular functions, immune response, and the synthesis of hormones and neurotransmitters. Adequate vitamin intake through diet or supplementation is therefore crucial for maintaining homeostasis and preventing disorders associated with deficiency.

Vitamins can exert regulatory effects through epigenetic mechanisms, acting as cofactors for specific enzymes, modulating gene expression, and influencing histone modifications or DNA methylation. They have also been shown to influence the expression of microRNAs (miRNAs), small non-coding RNAs that modulate gene expression at the post-transcriptional level [2]. Although miRNA regulation has been demonstrated for several vitamins, most existing studies have been conducted in disease-specific or clinical contexts and typically rely on small sample sizes, limiting generalizability.

B vitamins are water-soluble and play a crucial role in energy metabolism, nervous system function, red blood cell formation, DNA synthesis, and immune response. Vitamin B12 (cobalamin) contributes to the regulation of specific immune cell populations [3]. Extremely high serum B12 concentrations have been associated with increased risk of various cancer types [3], likely due to its involvement in one-carbon metabolism, DNA synthesis, and energy metabolism [4]. Only a limited number of studies have investigated the influence of vitamin B12 on miRNA expression, mainly in the context of maternal dietary supplementation during pregnancy [5, 6]. Folate (vitamin B9) is essential for cell proliferation and erythropoiesis and, similar to vitamin B12, serves as a key regulator of one-carbon metabolism. Previous research has predominantly investigated associations between folate and miRNAs in various cancers [7–9] or in relation to neural tube defects in early pregnancy [10]. Both vitamins are essential during pregnancy as they support fetal brain and nervous system development and prevent neural tube defects [11]. They are also supporting neuropsychiatric health [12] and the immune system [3]. Vitamin B12 and folic acid are metabolically linked through one-carbon metabolism, which is essential for DNA synthesis and cell division. Vitamin B12 serves as a cofactor for methionine synthase, enabling the conversion of folate into its active form required for nucleotide synthesis [13].

In the present study, we aimed to expand the current knowledge by investigating the association between circulating serum levels of folic acid (vitamin B9), and vitamin B12 and plasma levels of circulating miRNAs in the population-based cohort SHIP-TREND-0 (*N* = 792). Without focusing our analysis on a specific disease, we seek to identify miRNAs whose plasma circulating levels are altered by the vitamins in a population-based setting and to further characterize the biological processes and disease pathways in which these miRNAs are known to be involved. This approach may contribute to a deeper understanding of the health-supporting roles of the analyzed vitamins and molecular consequences of vitamin deficiency.

## Methods

### Study population

The investigations in the Study of Health in Pomerania (SHIP) were carried out in accordance with the Declaration of Helsinki, with all participants providing written informed consent. The survey and study methods were approved by the institutional review boards of the University of Greifswald.

SHIP is a population-based project from the northeast of Germany, designed to evaluate the prevalence and incidence of common diseases and their associated risk factors in the population [14]. Between 2008 and 2012, the SHIP-TREND-0 sample (referred to as TREND-0) was recruited, comprising 4420 participants. These individuals underwent a standardized computer-assisted personal interview, providing detailed information on sociodemographic and lifestyle factors. Additionally, several OMICS profiling approaches and biomarker analyses have been carried out with the available various biofluid samples. In a subsample of 792 TREND-0 participants, data on plasma-circulating miRNA levels were available. Medications taken within the past 14 days were classified according to the Anatomical Therapeutic Chemical (ATC) classification system.

### Covariates

Blood samples were collected from peripheral blood using ante-cubital venipuncture. The blood was taken in the supine position throughout the whole year. Plasma was obtained by centrifugation at 3570 ×g for 5 min at 18 °C, and aliquots were frozen at − 80 °C. Various blood-based biomarkers like the whole blood count with absolute levels of white blood cells (WBC), lymphocytes, neutrophils, platelets (PLT) (in Gpt/l), and red blood cells (RBC) (Tpt/l) are available. High-sensitivity C-reactive protein (hs-CRP) concentrations were determined in serum by nephelometry on the Dimension VISTA (Siemens Healthcare Diagnostics, Eschborn, Germany). Plasma fibrinogen concentrations were measured using Clauss method assessed by coagulation analyzers (BCS-XP; Siemens Healthcare Diagnostics, Germany). Body mass index (BMI) was defined as (weight in kg)/(height in m)^2^, smoking habits were divided into never, former, and current smokers. Alcohol intake was divided into low, medium, and high based on the monthly frequency of alcohol intake. Serum cystatin C concentrations were measured by the particle-enhanced immunonephelometry (N Latex Cystatin C kit, Dade Behring, Marburg, Germany) on the Behring Nephelometer II analyzer (Dade Behring, Marburg, Germany).

### Vitamin measurement

Serum folic acid and vitamin B12 were measured after one freeze-thaw cycle using solid-phase, competitive chemiluminescent enzyme immunoassay analyses on a Siemens IMMULITE^®^ 2000 automated, quantitative immunoassay analyzer (Siemens Healthcare Diagnostics, Eschborn, Germany). Vitamin B12 was measured within the range of 64 to 2508 pg/ml and folic acid from 1.8 to 100 ng/ml. Vitamin B12 deficiency was defined as < 250 pg/ml and folic acid deficiency as < 5 ng/ml. Folic acid measurement includes the inactive and more stable form of folate as well as well as active forms [15]. This measurement is a broad indicator of folate availability of the organism.

### miRNA measurement and preprocessing

For approximately 1000 participants in TREND-0, additional omics data, including SNP-based genetic data and gene expression profiles, were available. The three miRNA batches were selected from this subsample. Batches 1 and 2 were randomly selected to be representative for this subsample. Batch 3 was originally recruited as a psychiatric study and is enriched for individuals with a history of childhood trauma. In summary, circulating miRNA levels from plasma were measured in a subsample of 813 TREND-0 participants, divided into three distinct batches (371, 337 and 105 subjects). To account for technical variability in each batch, synthetic spike-ins (UniSp2, UniSp4, and UniSp5) were added prior to the extraction of plasma circulating miRNAs. Before using RNA samples for miRNA profiling, the presence of spike-ins (UniSp2, UniSp4, UniSp5), yield of typical plasma miRNAs, absence of PCR inhibitors (UniSp6, Cel-miR-39, UniSp3), as well as hemolysis in the samples, was assessed by use of a microRNA QC PCR Panel V1.M (Qiagen, Hilden, Germany). Samples failing quality control were excluded from further processing. For RT-qPCR-based miRNA analysis, the Serum/Plasma Focus microRNA PCR-Panel (Qiagen, Hilden, Germany) V3.M and V4.M were employed, covering 179 miRNAs. Resulting Ct values below 37 were considered for quantification and Ct values above 37 were treated as missing because they were considered to be too close to the detection limit of the assay. The remaining Ct values were normalized to the lower quartile per sample. After quality control, technical effects were regressed out, and the resulting residuals were used as independent variables in subsequent analyses (see Supplementary Information). MiRNA preprocessing followed our previously published study [16]. A miRNA was included in the analysis if at least 100 subjects had valid measurements for that miRNA (see Table S1 for a list of all miRNAs used), which resulted in 181 miRNAs for analysis. To account for hemolysis while preparing the plasma probes, we calculated the ∆Ct(miR-23a - miR-451) as hemolysis indicator that is used as covariate in the statistical regression models [17].

### Statistical analyses

Subject characteristics of the final study sample were summarized using medians and quantiles (25%, 75%) for continuous variables, and counts with percentages for categorical variables. Differences between males and females were assessed using Chi^2^ test for categorical data and non-parametric tests for dimensional data as most of these variables were not normally distributed. Various regression analyses were conducted to examine associations between different vitamin levels, blood cells, and circulating miRNAs in the general population.

*Direct effects of vitamins on miRNA levels*: Linear regression models were used to investigate the association between vitamin levels (predictor) and miRNA residuals (outcome). Analyses were adjusted for seasonality, age (both modeled as natural spline), sex, and BMI, but also for PLT, miRNA batch, and miRNA-based hemolysis indicator.

$$\begin{aligned}&miRNA\sim vitamin+season+\\&age+sex+PLT+batch+hemolysis+BMI\end{aligned}$$

*Direct effects of vitamins on blood cell count (BCC) and inflammation markers*: Linear regression models were used to investigate the association between vitamin levels (predictor) and main blood cell counts (WBC, RBC, PLT) as well as markers of inflammation (CRP, fibrinogen) as outcome. Analyses were adjusted for seasonality, age (both modeled as natural spline), sex, and BMI.

$$BCC \sim vitamin+season+age+sex+BMI$$

Covariates were selected based on our prior findings [16, 18] or relevant literature [19, 20]. Vitamin levels were analyzed as either a continuous variable (log_2_-transformed due to skewness) or as a categorical variable (deficiency vs. non-deficiency based on medical cutoffs).

Multiple testing correction across the miRNA dataset was performed using Benjamini-Hochberg (BH) method. For significant miRNAs, regression residuals were manually inspected for normality. For the blood cell count, BH-correction was applied. All analyses were conducted in R version 4.3.0 (https://cran.r-project.org/). Significant miRNAs were followed up analyzing their contribution in different blood cell types and their involvement in biological processes and previous associations with diseases using publicly available databases.

## Results

The final study sample consisted of 792 subjects with complete data for all variables included in the main analyses. The sample included individuals aged 21–79 of which 52% were females. Participants had a median BMI of 27 ranging from 16.7 to 48.1. Among all participants 16% were classified as vitamin B12 deficient (< 250 pg/ml), and 11% as folic acid deficient (< 5 ng/ml). Significant differences between males and females were observed for BMI, platelet count, RBC, WBC, smoking status, alcohol intake, and inflammatory markers (Table 1). The correlation between both vitamins was *r* = 0.22.

**Table 1:** Sample characteristic (*N* = 792) of all study subjects with complete data for vitamins and blood cells as well as available miRNA data in TREND-0

	Males (*N* = 378, 47.8%)	Females (*N* = 414, 52.2%)	Comparison
**Age (in years)**	49 (37, 60)	50 (40, 59)	*P* = 0.83
**BMI**	27.5 (25.0, 29.9)	26.1 (23.4, 30.0)	*P* = 0.004
**PLT (Gpt/l)**	209 (184, 238)	238 (206, 276)	*P* < 0.001
**Fibrinogen (g/l)**	2.7 (2.2, 3.2)	3.2 (2.6, 3.5)	*P* < 0.001
**CRP (mg/l)**	0.95 (0.52, 1.79)	1.32 (0.67, 3.00)	*P* < 0.001
**WBC (Gpt/l)**	5.3 (4.7, 6.3)	5.6 (4.8, 6.6)	*P* = 0.04
**RBC (Tpt/l)**	4.9 (4.6, 5.1)	4.5 (4.2, 4.7)	*P* < 0.001
**Vitamin B12 (pg/ml)**	342 (277, 422)	343 (275, 435)	*P* = 0.63
**Folate (vitamin B9) (ng/ml)**	9.5 (6.4, 13.2)	9.7 (6.3, 13.9)	*P* = 0.43
**Smoking status**			*P* < 0.001
** Never**	119 (31.5%)	193 (46.6%)	
** Former**	173 (45.8%)	124 (30.0%)	
** Current**	86 (22.7%)	96 (23.2%)	
**Alcohol intake**			*P* < 0.001
** Low**	21 (5.6%)	37 (8.9%)	
** Medium**	171 (45.2%)	310 (74.9%)	
** high**	186 (49.2%)	67 (16.2%)	
**miRNA Batch**			*P* < 0.001
** 1**	181 (47.9%)	177 (42.8%)	
** 2**	170 (45.0%)	161 (38.9%)	
** 3**	27 (7.1%)	76 (18.3%)	

For metric variables mean, quantiles (25%, 75%) are reported; for categorical variables numbers and percentages are given. Significant differences were tested with Wilcoxon test for metric variables and Chi^2^ test for categorical data. BMI: Body Mass Index, PLT: platelet count, CRP: C-reactive protein, WBC: white blood cell count, RBC: red blood cell count. Smoking status was missing for one subject, CRP levels for two subjects, fibrinogen levels for 8 subjects

### Association between vitamins and miRNAs

**Fig. 1:**
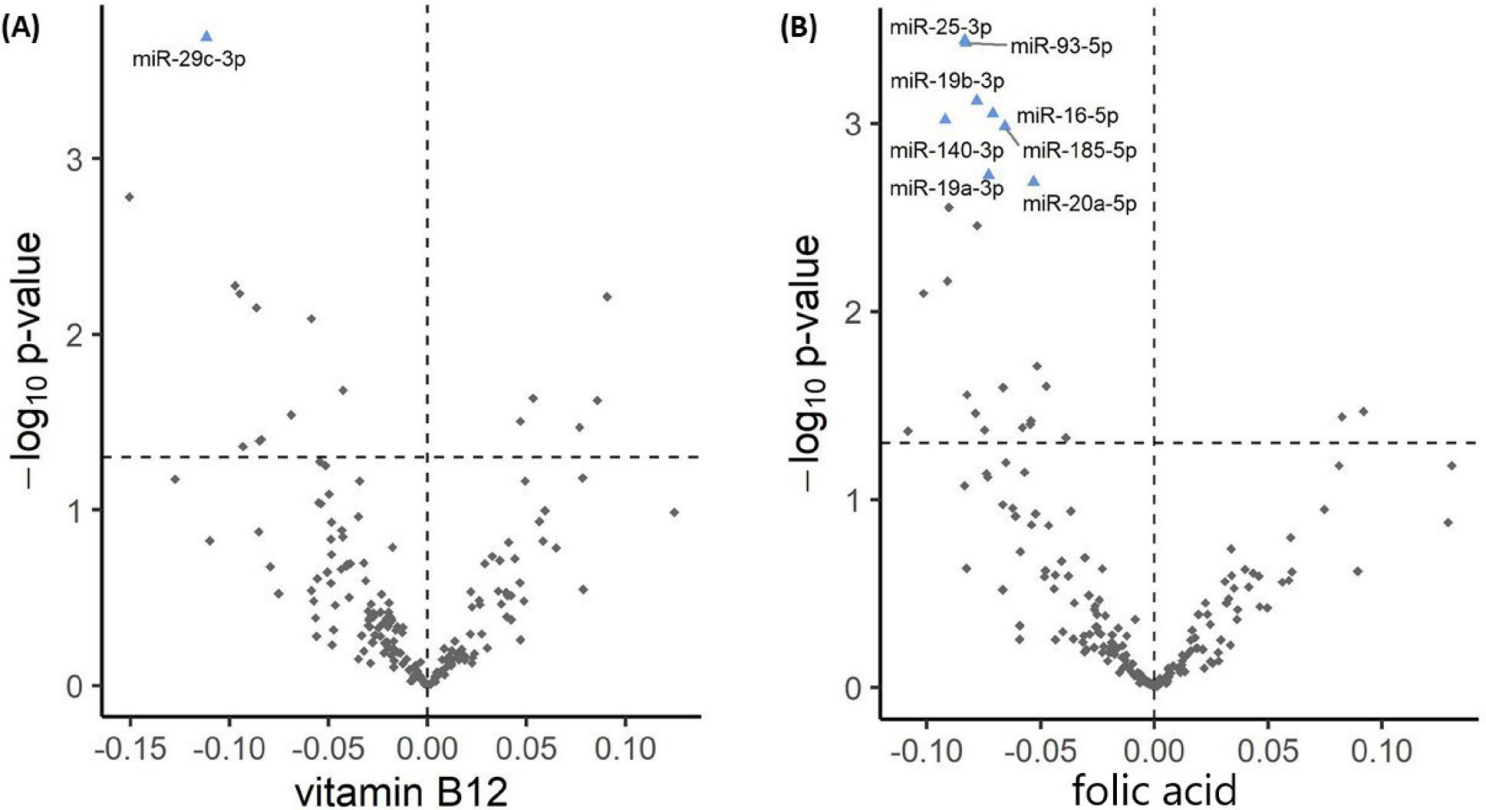
Associations between circulating miRNAs and vitamin B12 levels (**A**) and folic acid levels (**B**), respectively. Significantly associated miRNAs (after correction for multiple testing) are highlighted and named. On the x-axis standardized estimates are shown. For the miRNAs ∆CT-values are available and positive estimates (β_∆Ct_ > 0) indicate a negative association (since larger ∆Ct values indicate lower miRNA levels) and vice versa

Associations between vitamin B12 and folic acid on 181 miRNAs were tested in 792 individuals from TREND-0. Significantly associated miRNAs have been found for vitamin B12 as well as for folic acid (Fig. 1, Supplementary Tables S1/2). Vitamin B12 was significantly associated with higher levels of miR-29c-3p (*p*_*BH*_ = 0.037). For folic acid, eight miRNAs were positively associated after BH correction, miR-25-3p, miR-93-5p, miR-19a-3p, miR-19b-3p, miR-16-5p, miR-140-3p, miR-185-5p, and miR-20a-5p (minimal *p*-value *p*_*BH*_ = 0.031). Additional adjustment for white and red blood cell count did not affect the significance of the association of the previously identified miRNAs. Thus, there was probably no mediating effect of these blood cells on the miRNA levels. There was also no strong overlap between the nominal significant miRNAs from both analyses although both vitamins are biologically linked. Sex stratified analyses revealed no significant differences between males and females in the associations of vitamin and miRNA levels (Supplementary Tables S3-6).

Separating individuals into deficient and non-deficient for the different vitamins revealed that vitamin B12 deficiency was associated with additional miRNAs. We observed 129 subjects with vitamin B12 deficiency and 90 with folic acid deficiency. Vitamin B12 deficiency was associated with miR-660-5p, miR-22-3p, miR-222-3p, miR-29b-3p, miR-29a-3p, as well as previously identified miR-29c-3p which showed higher abundance in subjects with normal vitamin B12 levels. For folic acid deficiency no significant miRNAs emerged (Supplementary Tables S7-8). Results were also robust against additional adjustment for smoking status, alcohol consumption, or serum cystatin C as marker for kidney function.

### Enrichment in blood cell types

**Fig. 2:**
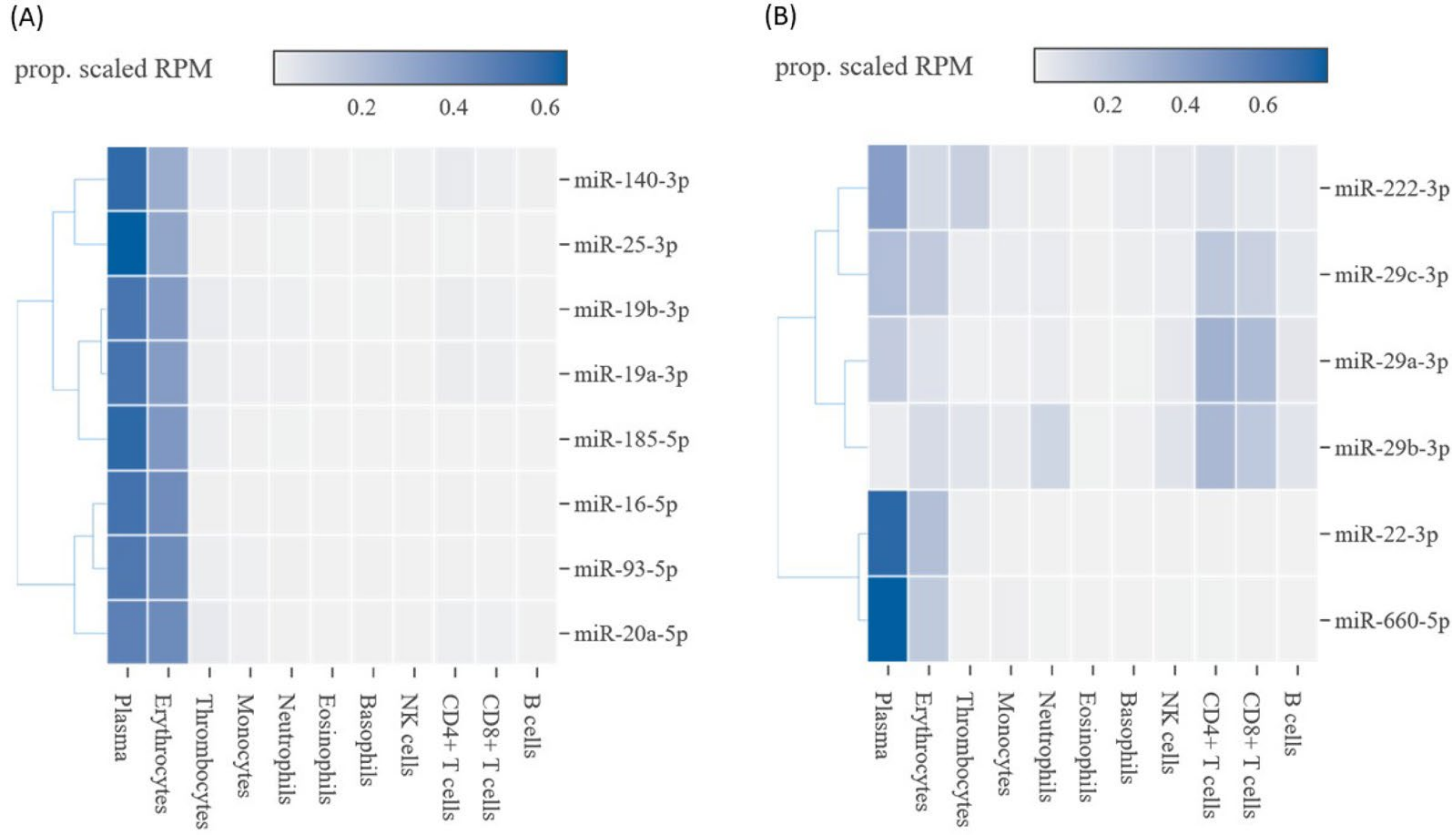
Relative contribution of blood components to the levels of the miRNAs significantly associated with folic acid (**A**) and vitamin B12 (**B**) deficiency in whole blood. Data from the miR-Blood database [21] using scaled proportions of the reads per million (RPM). miRNAs significantly associated with folic acid are mainly found in plasma and red blood cells whereas miRNAs associated with vitamin B12 are found in plasma, red blood cells and miR-29 also at a high amount in T cells

According to the miR-Blood database (https://mir-blood.com/ [21]), , all miRNAs significantly associated with folic acid are predominantly located in plasma and red blood cells (Fig. 2A). The significantly associated miRNAs for vitamin B12 showed a mixed pattern with miRNAs located in plasma, red and white blood cells (Fig. 2B). Associations between main blood cell components (RBC, WBC, PLT) and inflammation markers (CRP, fibrinogen) with the vitamins in our data revealed that folic acid was inversely associated with RBC (β_std_RBC_ = -0.09, *p*_*BH*_ = 0.008) and WBC (β_std_WBC_ = -0.12, *p*_*BH*_ = 0.005) and vitamin B12 positively with RBC (β_std_RBC_ = 0.075, *p*_*BH*_ = 0.05). Significant associations with PLT were not observed. Folic acid also revealed an inverse association with markers of inflammation which slightly missed significance (β_std_Fib_ = -0.07, *p*_*BH*_ = 0.078; β_std_CRP_ = -0.06, *p*_*BH*_ = 0.08).

Since both vitamins were associated with RBC, and vitamin-associated miRNAs are predominantly located in RBCs, we tested if the significantly associated miRNAs were also associated with RBC count in our sample. None of the miRNAs showed a significant association with RBC count, and thus no evidence for a possible mediating effect of the miRNAs on the path from vitamin levels to RBC count. As RBC count only reflects the quantity of RBCs and not the quality, results should be treated with caution.

### Enriched pathways and diseases

**Fig. 3:**
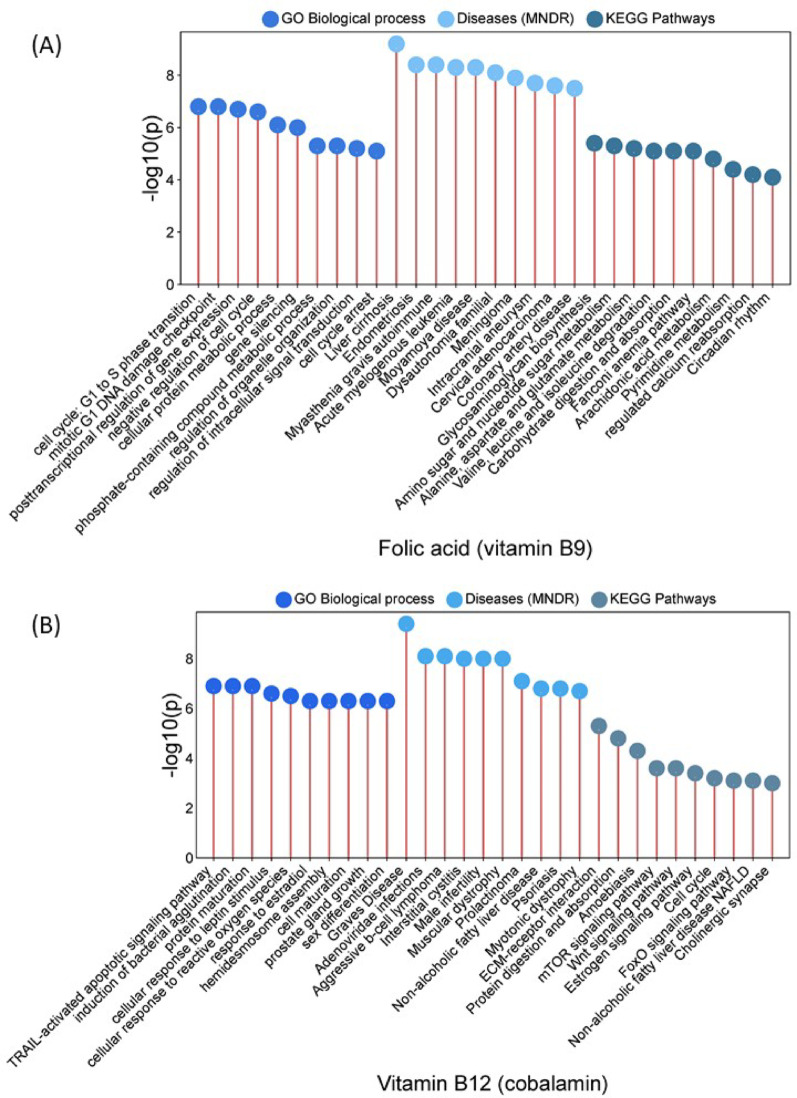
Results of the over-representation analyses for the significantly associated miRNAs for folic acid and vitamin B12 using the miEAA tool. Top 10 of the three categories “GO biological processes”, “Diseases” (using the MNDR – mammal ncRNA-disease repository; [32]), and “KEGG Pathways” (Kyoto Encyclopedia of Genes and Genomes). (**A**) results for the eight miRNAs significantly associated with folic acid, (**B**) results for the six miRNAs significantly associated with vitamin B12

In order to identify biological systems associated with the significant miRNAs, we used the miEAA tool (miRNA Enrichment Analysis and Annotation, https://ccb-compute2.cs.uni-saarland.de/mieaa/; [22]) that looks for over-representation of miRNA target genes in biological pathways. For the eight miRNAs associated with folic acid, significant over-represented processes involved cell replication, cell-cycle regulation, DNA synthesis, metabolic processes, gene expression, and apoptosis (Fig. 3A). Moreover, folate metabolism and erythropoiesis were over-represented for statistically significant miRNAs (Table S9). Further, an over-representation was also observed for the folic acid associated miRNAs and specific diseases based on data from MNDR (mammal ncRNA-disease repository). These diseases included liver cirrhosis, endometriosis, coronary artery disease, and different types of cancer. Significant results for the 6 miRNAs associated with vitamin B12 also revealed over-representation for erythropoiesis, cell-cycle regulation, and cell maturation. Over-represented diseases include Graves’ disease, muscular dystrophy, male infertility, and non-alcoholic fatty liver disease (Table S10).

## Discussion

In this manuscript, we investigated the linear relationship between serum vitamin levels for folic acid, and vitamin B12 as well as vitamin deficiency and miRNA abundance. In addition, we tried to link the identified miRNAs to regulated biological mechanisms and disease outcomes. Significantly associated miRNAs were revealed mainly for folic acid levels as well as for vitamin B12 deficiency, with no overlap. Our findings provide evidence for an effect of folic acid and vitamin B12 on plasma miRNA levels which was not influenced by existing associations among the vitamins and blood cell components, especially red and white blood cell count. The miRNAs identified were predicted to have an impact on various biological functions including cell proliferation, erythropoiesis, DNA synthesis, cellular metabolic processes, or fertility and pregnancy outcomes in line with the observed associations with blood cell count. Thus, the significant miRNAs might be involved in the complex process of vitamin induced cell proliferation and erythropoiesis by activating different miRNAs.

All eight miRNAs associated with serum folic acid levels were positively associated with the vitamin level. Among them, seven miRNAs have previously been identified in association with folic acid metabolism or related conditions. The cellular miRNA miR-25-3p is targeting the *DHFR* gene which is a key player in folate metabolism [23]. miR-25-3p has also been found to play a role in cell proliferation as it promotes tumor growth [24, 25]. In a study by Lee and colleagues, genetic variation in the MIR25 gene was found to be associated with recurrent implantation failure [26] which is supported by a study showing the benefit of folate on assisted reproduction and reproductive success [27]. This also reflects the strong impact of folate in pregnancy including the prevention of neural tube defects (NTD), anemia, and preterm birth. Also miR-185-5p was identified as a hub gene in folate metabolism and is acting as tumor suppressor gene [28]. Again, miR-185-5p was identified in relation with pregnancy as it was suggested as predictive marker for recurrent pregnancy loss suggesting an inflammation-mediated mechanism through VEGF [29]. In another study related to NTD, folate intake had a positive effect on NTD through epigenetic downregulation of miR-185-5p and introducing methylation changes [30]. Further, the miR-20a-5p was upregulated in brain tissue of low folate-induced NTD [31]. The miRNAs miR-19a-3p and miR-19b-3p both belong to the miR-19 family which has been found to regulate tissue development and homeostasis [32]. The miRNA family also plays a role in cell differentiation and proliferation including neurons or liver cells [33]. In addition, these miRNAs show associations with spinal cord injury and neuropathic pain and have been found to change in response to folic acid [34] which prevents down-regulation of these miRNAs [32, 35]. Also miR-16-5p has been found to interact with folate on the risk for pancreatic cancer [7] and also to suppress glioma cell proliferation by inhibiting the cell cycle and promoting apoptosis [36]. The miR-140-3p is involved in tissue repair after cerebral ischemia where vitamins such as folate and vitamin B12 act as cofactors [37]. It was also found to be involved in palate formation and cleft palate [38] which is known to also be associated with folate intake of the mother during pregnancy [39].

Similar to folic acid all six miRNAs significantly associated with vitamin B12 and B12 deficiency showed a positive association. Although data on the effects of vitamin B12 on miRNA expression are rare, some miRNAs could be linked to vitamin B12 and related conditions. Vitamin B12 deficiency is generally characterized by hematological or neurological problems [40]. All three miRNAs of the miR-29 family (29a/b/c) were upregulated in case of higher vitamin B12 levels in our sample. These miRNAs are involved in the regulation of cell differentiation, showing antifibrotic effects and regulating collagen expression and general aging phenotypes [41, 42]. On a broader level, they have also been associated with neurological phenotypes [43] and a neurodegenerative diseases [44]. Chawrylak and colleagues introduced miR-22-3p as a predictor of nutrition deficits in head and neck cancer patients [45] as miR-22-3p has been associated with various metabolic and nutrition processes. This malnutrition was defined via BMI cutoffs and underweight individuals showed lower miR-22-3p expression. MiR-222-3p has directly been linked to vitamin B12 levels in pregnant woman [46]. Both miRNAs, miR-22-3p and miR-222-3p, are possible candidate biomarkers for frontotemporal dementia [47]. The other miRNAs were previously identified in association with different tumors but without a direct link to vitamin B12 effects [48].

In addition to the effects of individual miRNAs, pathway enrichment analyses of folic acid and vitamin B12 associated miRNAs revealed strong connections to vitamin metabolism. Both vitamins play essential roles in one carbon metabolism and hematopoiesis [49, 50]. Fibrinogen associated miRNAs were predominantly over-represented in neurological disorders (Moyamoya disease, Myasthenia gravis, dysautonomia familial) as well as in conditions related to female reproduction (endometriosis, cervical adenocarcinoma). In contrast, vitamin B12 associated miRNAs were mainly linked to immune related disorders (Graves’ disease, psoriasis, adenoviridae infections), muscular dystrophy, and male infertility. Vitamin B12, similar to folate, has been suggested to play a role in both male and female infertility [51]. Nearly all significant miRNAs associated with both vitamins have been reported in the context of pregnancy related conditions. These include gestational diabetes [52, 53], preterm birth [54, 55], pregnancy-induced hypertension [56, 57], and fetal growth restrictions [58]. These findings suggest that folic acid and vitamin B12 may have broader implications for healthy pregnancy than previously suggested [59, 60] with miRNAs potentially acting as biological mediators of these effects on the regulatory level. Emerging evidence indicates that miRNAs may serve as biomarkers for pregnancy-related complications [57, 61]. Zhou et al. investigated the diagnostic potential of miR-25-3p for predicting hypertensive disorders during pregnancy [57] and reported that increased miR-25-3p levels were associated with hypertension and preeclampsia. Furthermore, a review analyzing miRNAs associated with gestational diabetes identified upregulation of the three folic acid associated miRNAs miR-16-5p, miR-19a-3p, and miR-19b-3p [62]. Additionally, miR-29a-3p and miR-29b-3p, both associated with vitamin B12, have been explored as potential biomarkers for gestational diabetes [63].

Our results are limited by the availability of an independent replication sample. Findings from the general population could not be transferred to a clinical setting regarding fertility and pregnancy outcomes which should be tested in further studies. The miRNA data in our sample is semi-quantitative and we cannot make conclusions for absolute values. Further studies should use quantified miRNA levels to define cutoffs for clinical decision making. Moreover, the subsample with available miRNA data is healthier than the full SHIP-TREND-0 study sample which hinders generalizability. In addition, folic acid can only be interpreted as a proxy for the current levels of active folate in the body.

## Conclusions

In conclusion, we confirmed that several miRNAs are altered in association with specific vitamin levels, particularly folic acid and vitamin B12, in the general population. The significantly associated miRNAs reflect the established metabolic and functional roles of these vitamins in human health and especially in adverse pregnancy outcomes. These miRNAs exhibit a pronounced over-representation in processes related to cell proliferation and associated conditions, including hematopoiesis and various types of cancer. Furthermore, a substantial impact on human fertility and pregnancy was reported for the significant miRNAs with first attempts to use them as biomarkers, highlighting miRNAs as potential targets for clinical applications. Further investigations should focus on exploring causal relationships between miRNAs and these vitamins and their causal relationship towards pregnancy outcomes.

## Supplementary Information

Below is the link to the electronic supplementary material.

Supplementary Material 1

## Data Availability

The data of the SHIP study cannot be made publicly available due to informed consent of the studyparticipants, but it can be accessed through a data application form available at http://fvcm.med.uni-greifswald.de/ for researchers who meet criteria for access to confidential data.

## Abbreviations

miRNA: micro–RNA
RBC: Red blood cells
WBC: White blood cells
PLT: Platelet count
CRP: C–reactive protein
BMI: Body mass index
NTD: Neural tube defects
